# Expression of the zebrafish intermediate neurofilament Nestin in the developing nervous system and in neural proliferation zones at postembryonic stages

**DOI:** 10.1186/1471-213X-7-89

**Published:** 2007-07-25

**Authors:** Julia Mahler, Wolfgang Driever

**Affiliations:** 1 Developmental Biology Department, Institute of Biology I, University of Freiburg Hauptstrasse 1, D-79104 Freiburg, Germany

## Abstract

**Background:**

The intermediate filament Nestin has been reported as a marker for stem cells and specific precursor cell populations in the developing mammalian central nervous system (CNS). Nestin expressing precursors may give rise to neurons and glia. Mouse *nestin *expression starts at the onset of neurulation in the neuroectodermal cells and is dramatically down regulated when progenitor cells differentiate and become postmitotic. It has been reported that in the adult zebrafish (*Danio rerio*) active neurogenesis continues in all major subdivisions of the CNS, however few markers for zebrafish precursors cells are known, and Nestin has not been described in zebrafish.

**Results:**

We cloned a zebrafish *nestin *gDNA fragment in order to find a marker for precursor cells in the developing and postembryonic brain. Phylogenetic tree analysis reveals that this zebrafish ortholog clusters with Nestin sequences from other vertebrates but not with other intermediate filament proteins. We analyzed *nestin *expression from gastrula stage to 4 day larvae, and in post-embryonic brains. We found broad expression in the neuroectoderm during somitogenesis. In the larvae, *nestin *expression progressively becomes restricted to all previously described proliferative zones of the developing and postembryonic central nervous system. *nestin *expressing cells of the forebrain also express PCNA during late embryogenesis, identifying them as proliferating precursor or neural stem cells. *nestin *is also expressed in the cranial ganglia, in mesodermal precursors of muscle cells, and in cranial mesenchymal tissue.

**Conclusion:**

Our data demonstrate that in zebrafish, like in mammals, the expression of the intermediated neurofilament *nestin *gene may serve as a marker for stem cells and proliferating precursors in the developing embryonic nervous system as well as in the postembryonic brain.

## Background

The intermediate filament Nestin has been reported as a marker for stem cells as well as precursor populations of specific cell types in the developing mammalian central nervous system (CNS) giving rise to both neurons and glia [1,2]. Nestin is classified as type IV neurofilament, which together with microfilaments and microtubules constitute a major component in the cytoskeleton. In contrast to other more general cytoskeletal elements, intermediate filaments are expressed in a cell type specific manner, and major differentiation steps are marked by the transition from one intermediate filament type to another. With the onset of neurulation neuroectodermal cells start to express *nestin*. The expression is dramatically down regulated when progenitor cells differentiate and become postmitotic [2-4], reviewed in [5].

*nestin *mRNA also has been reported to be expressed in the developing myotome [6] and skeleton muscle precursors [2,6], as well as in mesenchymal pancreatic cells [7], the intestine [8], and cranial ganglia [9].

While Nestin has been investigated extensively in mammalian systems, including rat [2], mouse [10], and human [11], as well as in chick [12], it has not been reported for fish so far. Zebrafish have evolved as a genetic and experimental model organism that is ideally suited to study basic principles of neural development [13-15]. It has been reported that in the adult zebrafish active neurogenesis continues in all major subdivisions of the CNS [16]. Thus, zebrafish serve as an excellent model for studying neural stem cells and neural regeneration. In detailed BrdU incorporation studies, distinct proliferation zones have been identified in all subdomains of the zebrafish brain along the rostrocaudal CNS axis [16-18]. It appears that, like in mammals, neuroblasts are also continuously generated in neurogenic niches in the subependymal/subventricular regions of the brain.

While BrdU incorporation studies may identify neural stem cells, these experiments are not easily integrated into most experimental setups. Other markers used to identify zebrafish neural stem cells include: PCNA [19], MCM5 [15,20], and anti-phospho-histone antibodies. However, these markers are not restricted to neural proliferating cells. To identify a specific marker for neural stem cells and precursors, we cloned and analyzed the expression of a zebrafish Nestin ortholog.

## Results

### A zebrafish *nestin *ortholog

**Figure 1:**
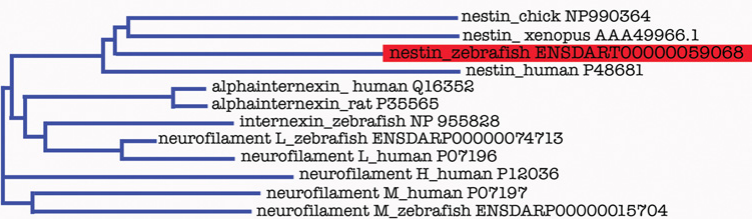
**Phylogenic analysis of nestin protein**. The predicted *nestin *sequence (Ensembl) was translated into protein sequence and compared with other nestin proteins and intermediated neurofilaments of human, frog, chick, rat, and zebrafish. The analysis was performed with VectorNTI software (Neighbor joining method). For each protein either the NCBI, SwissProt or Ensembl ID numbers are noted.

In order to find a marker for precursor cells in the developing zebrafish brain we analyzed *nestin *(*nes*) homologous genes in zebrafish. We compared *nestin *sequences from various different species and performed a blast search of zebrafish genomic sequences to identify whether there are duplicated zebrafish *nestin *genes. We identified a single *nestin *ortholog with the ENSEMBL gene number ENSDAR00000040236 [21]. We compared the predicted mRNA as well as other zebrafish intermediate filaments with protein sequences from other species. Phylogenetic tree analysis revealed that the predicted zebrafish Nestin protein clusters with mouse, human, and chicken Nestin, but not with the other intermediated neurofilaments (Fig. 1). Using PCR on genomic DNA we cloned a 704 bp *nestin *fragment covering the second exon, we generated antisense probes, and investigated *nestin *expression in the developing zebrafish brain.

### Expression of *nestin *during zebrafish somitogenesis and in neuronal tissues

We assayed the *nestin *expression pattern by *in situ *hybridization with an antisense RNA probe in zebrafish embryos and larvae staged from 60 % epiboly up to 96 hours post fertilization (hpf). We also determined *nestin *expression in sections of the post-embryonic zebrafish brain at 28 dpf.

**Figure 2:**
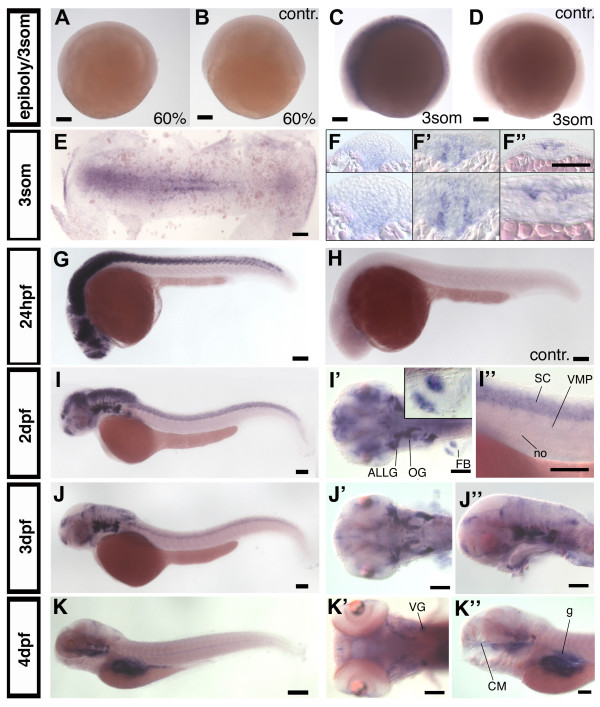
***nestin *expression in zebrafish development**. (A-D): *nestin *expression pattern during gastrula and early somitogenesis stages, (A) *nestin *is not expressed at 60% epibloy stage, (B) sense control. (C, D): First expression is detectable at 3 somite stage. (E, F-F"): Flat mount and cross sections of a 3 somite embryo. (G, sense control in H): Lateral view of 24 hpf embryo, *nestin *is expressed widely in CNS. (I, I'): 2 dpf, lateral view (I), dorsal view (I') *nestin *is expressed in head ganglia and fin buds; insert in (I') shows higher magnification of a fin bud; (I") higher magnification of the spinal cord. *nestin *expression in the ventral root may correlate with glia or neuroblasts. (J-J"): At 3 dpf *nestin *expression becomes more restricted to proliferation zones, (J) lateral view, (J', J") higher magnifications, dorsal (J') and lateral (J") view. (K-K"): 4 dpf: *nestin *expression in the CNS is almost completely restricted to proliferation zones. Further expression is detected in the cranial ganglia, the gut and the craniofacial mesenchyme (K"). *Abbreviations: *ALLG: anterior lateral line ganglion; cm: craniofacial mesenchyme; FB: fin bud; g: gut; OG: octaval ganglion; no: notochord; SC: spinal cord; VG: vagal ganglion; VMP: ventral motoneuron precursors. (A-E, G-K"): anterior left, animal pole up, (F-F"): cross sections, dorsal up. Scale bars: 100 μm, except K: scale bar: 200 μm.

*nestin *expression was not detected before the 3 somite stage (10–11 hpf) (Fig. 2A,B). Earliest expression of *nestin *was detected during mid-neurulation (Fig. 2C–F"). At this stage *nestin *is expressed in a domain laterally adjacent to midline cells. Control embryos processed with a sense RNA for *nestin *show no obvious staining at this stage (Fig. 2D). Serial sections reveal that adaxial mesoderm cells express *nestin *in the trunk, and in the posterior trunk *nestin *is expressed in the neural plate (Fig. 2F–F"). At 18 somite stage *nestin *is also expressed in premigratory and migrating neural crest cells (data not shown).

At 24 hpf *nestin *is widely expressed throughout the developing nervous system (Fig. 2G). A similar widespread expression has been reported for comparable stages of mouse development [1,3]. Zebrafish *nestin *expression is restricted to more defined regions of the CNS as the embryo further develops. We will now first describe the non-neuronal expression domains and later focus on the CNS expression.

**Figure 4:**
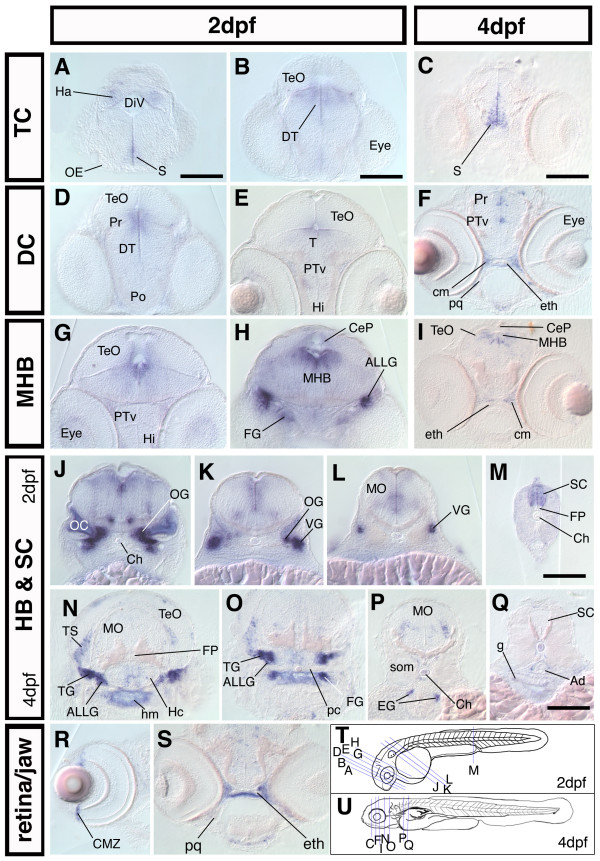
***nestin *is expressed in proliferative zones during the development of the CNS**. Cross sections of 2 dpf and 4 dpf embryos; dorsal up. (A-S): frontal and transversal cross section of *nestin *expression along the rostrocaudal axis of the brain and in the spinal cord reveals expression in major proliferative zones The relative rostrocaudal levels of each section are indicated in T and U. (A-I): sections at levels of forebrain and midbrain as well as midbrain-hindbrain boundary. (J-Q): sections at the level of HB and SC, showing *nestin *expression in the cranial ganglia and the HB, MO, SC. (R): cross section through the eye of a 4 dpf embryo, *nestin *is expressed in the CMZ; (S): cross section of a 4 dpf embryo at the level of the ethmoid plate, *nestin *is expressed in the craniofacial mesenchyme adjacent to developing cartilage tissue. (T, U): scheme of a 2 dpf (T) and a 4 dpf (U) zebrafish embryo, the levels of the cross sections shown in A-S are indicated by blue lines. *Abbreviations: *Ad: Aorta dorsalis; ALLG: anterior lateral line ganglion; CeP: cerebellar plate; Ch: chorda dorsalis; cm: craniofacial mesenchyme; CMZ: ciliary marginal zone; DiV: diencephalic ventricle; DT: dorsal thalamus; eg: enteric ganglia; eth: ethmoid plate; FG: facial ganglion; FP: Floor plate; g: gut; Ha: habenula; Hc: caudal hypothalamus; Hi: intermediate hypothalamus; hm: head mesenchyme; MHB: midbrain hindbrain boundary; MO: medulla oblongata; OC: otic capsule; OE: olfactory epithelium; OG: octaval ganglion; pc: parachordal cartilage; pq: palatoquadrate; Po: preoptic region; Pr: pretectum; PTv: ventral part of the posterior tuberculum; S: subpallium; SC: spinal cord; som: somites; T: midbrain tegmentum; TeO: optic tectum; TG: trigeminal ganglion; TS: torus semicircularis; VG: vagal ganglion. Scale bars: 100 μm.

Outside of the nervous system, mammalian *nestin *has been reported to be expressed in head mesenchyme and muscle precursors [6]. In contrast to *nestin *expression in mouse, zebrafish *nestin *was detected neither in developing somites nor in the myotome of mature somites. In fin buds *nestin *is expressed at 2 dpf in two stripes of mesoderm which will give rise to muscle tissue (Fig. 2I'). Further, from 4 dpf on *nestin *is expressed in craniofacial mesenchyme adjacent to the ethmoid plate (eth) and palatoquadrate (pq) (Fig. 2K"; Fig. 4F,I,S) and weakly in epithelial cells of the gut (Fig. 2K"; Fig. 4Q) [8].

### Expression of *nestin *in the central nervous system (CNS)

**Figure 3:**
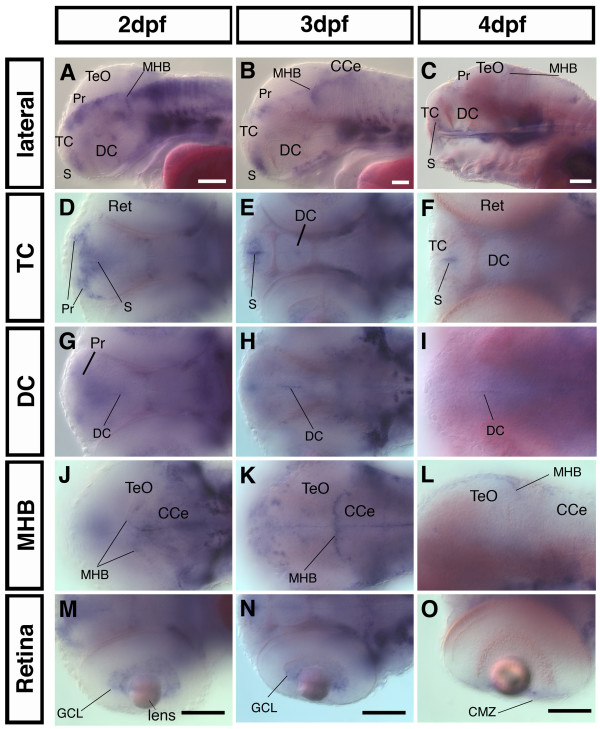
**In the developing CNS *nestin *expression becomes progressively restricted to proliferative zones**. Analysis of *nestin *expression in the head by whole mount *in situ *hybridization. (A-C): lateral views, anterior left, dorsal up, eyes were removed for better visualization of the expression pattern in the DC. A mid-saggital focal plane is shown (D-O, except L): dorsal views, anterior left, the dorsoventral level of the focal plane is indicated at left. (L): lateral view. *Abbreviations: *CCe: Cerebellum; CMZ: ciliary marginal zone; DC: diencephalon; GCL: ganglion cell layer; MHB: midbrain hindbrain boundary; Pr: pretectum; Ret: retina; S: subpallium: TC: telencephalon; TeO: optic tectum. Scale bars: 100 μm.

At 24 hpf *nestin *is expressed widely throughout the developing nervous system. Comparing *nestin *expression at 2 dpf, 3 dpf, and 4 dpf in zebrafish embryos reveals that *nestin *expression becomes progressively restricted to domains where the progenitor cell pools have been identified [18]: Ventricular zones (VZ) in the telencephalon (TC) and diencephalon (DC), the midbrain hindbrain boundary (MHB), and the ciliary marginal zone (CMZ) of the retina (Fig. 2G,I,J,K). From 2 dpf to 4 dpf, in the TC and DC, *nestin *expression starts to be restricted to the ventricular zones in the region of the subpallium and the ventral DC (Fig. 3A–I;). At 2 dpf *nestin *expression in the ventricular region of the DC is difficult to discern in whole mount ISH embryos because of the broad expression domain in the pretectal area (Fig. 3G). At 4 dpf the expression in the DC becomes more distinct (Fig. 3F). The pretectal *nestin *expression domain becomes also more restricted with further development of the nervous system and at 4 dpf it is restricted to very faint expression in a domain close to the ventricle (Fig. 3C). In the retina *nestin *expressing cells are initially detected in a broad zone including the ganglion cell layer (Fig. 3M–O). Similar to the diencephalon, *nestin *expression in the retina continuously regresses and by 4 dpf becomes restricted to the ciliary marginal zone, which constitutes the retinal proliferation zone [22,23] (Fig. 3O). Further posterior in the area of the MHB, the distribution of *nestin *positive cells also becomes more localized to the ventricle walls (Fig. 3G,H,I) from 1 dpf to 4 dpf. The MHB is clearly lined by a row of *nestin *expressing cells at 3 dpf (Fig. 3K). In the hindbrain initially broad *nestin *expression becomes restricted to cell populations adjacent to the ventricle and to some dorsal areas. Expression in the cerebellum also progressively decreases (Fig. 2J,J"; Fig. 3A–C).

Further we performed sections through 2 dpf and 4 dpf embryos to analyze in more detail the *nestin *expression pattern especially in regions, which were difficult to examine by transmitted light microscopy of whole-mount *in situ *hybridized embryos. In the TC *nestin *expression is restricted to ventricular zones (Fig. 4A). From 2 dpf to 4 dpf *nestin *expression in the area of the dorsal TC and the pretectal area is strongly down regulated (Fig. 4A–F). In the DC cells close to the ventricle express *nestin *predominantly in the dorsal part of the ventricular region of the DC (Fig. 4D,E). In parts of the intermediate hypothalamus *nestin *is expressed at 4 dpf (Fig 4N,O). Transversal sections at the level of the MHB through 2 dpf and 4 dpf zebrafish embryos revealed a strong *nestin *expression in this area (Fig. 4G–I) and correlates with regions of ongoing active neurogenesis [24]. Further, in the HB a cluster of cells bilaterally adjacent to the midline expresses *nestin *(Fig. 2I'; Fig. 4J). At 4 dpf expression in the hindbrain is almost completely absent, except for some scattered *nestin *expressing cells in the medulla oblongata (Fig. 4P). Sections as well as whole mount ISH revealed, that *nestin *is expressed throughout the spinal cord, except for the floorplate (Fig. 2I,I"; Fig. 4M). Zimmerman and colleagues [6] report that in the nestin:lacZ transgenic mice nestin:LacZ is not expressed in the floorplate and ventral midline. *nestin *mRNA is detectable in the ventral root of the spinal cord (Fig. 2I"). Mammalian motor neurons do not express *nestin *[6], but an antibody against Nestin protein (rat401) stains rat ventral root before axon outgrowth [1].

### Expression of *nestin *in the peripheral nervous system (PNS)

*nestin *in mammals is strongly expressed in ganglia of the posterior cranial nerves [9]. We also find strong expression at 2 dpf and 3 dpf in the octaval ganglion (ganglia of the n. octavus, VIII, vestibulocochlear nerve) (Fig. 2I'; Fig. 4J,K), the anterior and posterior part of the lateral line ganglia (associated with parts of VIIIth ganglion) (Fig. 2I'; Fig. 4H), the facial ganglia (n. facialis, VII) (Fig. 4H), and the ganglia of the nervus vagus (X) (Fig. 4K,L). The ganglia of the nervus vagus and the lateral line ganglia at this stage may contain a mixture of already differentiated cells and progenitor cells, with the latter situated more proximal to the midline than the differentiated cells. At 3 dpf *nestin *is still broadly expressed in cells of cranial ganglia (Fig. 2J). At 4 dpf the expression appears reduced (Fig. 2K',K"), but transversal sections through the head show that at 4 dpf *nestin *is still expressed in the trigeminal (V) ganglion, the octaval and lateral line ganglia, and the vagal ganglion (Fig. 4N,O), and data not shown). In the torus semicircularis (TS), a sensory nucleus which is the mesencephalic target of the octavolateralis-system [25], *nestin *expression can also be detected (Fig. 4N,O). Similar to mammals [8], at 4 dpf *nestin *expression is also detected in enteric ganglia of the peripheral nervous system (PNS) (Fig. 4P).

### *nestin *expressing cells co-express *pcna* in the forebrain during late embryogenesis

**Figure 5:**
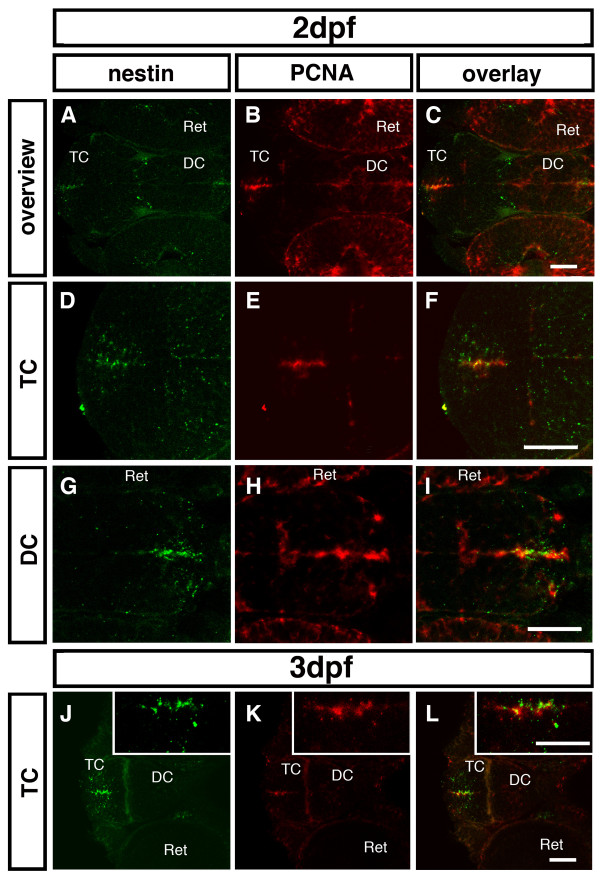
**Co-expression of *nestin *and *pcna *in the zebrafish forebrain**. Double fluorescent ISH was performed for 2 dpf (A-I) and 3 dpf (J-L) zebrafish embryos. *nestin *is shown in green (A, D, G, J); *pcna *in red (B, E, H, K); overlay (C, F, I, L). Inserted pictures in (J-L) show a higher magnification of the boxed areas. (A-C): Overview over the proliferation zones in the 2 dpf zebrafish forebrain. (D-F): Higher magnification of the ventricular zone of the TC shows co-expression. (G-H): Higher magnification of the subventricular zone in the ventral DC reveals co-expression of *nestin *and *pcna*. (J-L): At 3 dpf cells co-expressing *nestin *and *pcna *are detectable along the walls of the telencephalic ventricle. *Abbreviations: *DC: diencephalon; Ret: retina; TC: telencephalon. Scale bar: 50 μm.

Nestin has been reported as a marker for precursor populations of specific cell types in the developing mammalian CNS [1,2]. The expression of *nestin *in cells close to the ventricle throughout the CNS led to the assumption that also in zebrafish *nestin *is a marker for proliferating precursor cells. Therefore, we performed double fluorescent *in situ *hybridization to detect *nestin *and *proliferating cell nuclear antigen *(PCNA) expression to investigate whether *pcna *positive cells also co-express *nestin *in zones of proliferation. At 2 dpf we could detect areas with cells expressing both *nestin *and *pcna *in the proliferation zones of the forebrain (Fig. 5A–C). Cells expressing *nestin *and *pcna *are located along the TC ventricle (Fig. 5D–F) and in the ventricle walls of the ventral DC (Fig. 5G–I). Further, at 3 dpf co-expression was detectable in the subventricular zone of the TC (Fig. 5J–L). Thus, *nestin *expression appears to correlate with stem cell and precursor territories in the forebrain proliferation zones. However, expression domains of *nestin *in the peripheral ganglia do not correlate with *pcna *expression (data not shown), confirming that *nestin *is also expressed in postmitotic or differentiated cells in the peripheral nervous system.

### *nestin *expression in post-embryonic stem cell zones and migrating precursors

In order to investigate *nestin *expression in the post-embryonic zebrafish brain we examined *nestin *mRNA distribution in 28 dpf zebrafish brain. We performed whole brain ISH and generated serial transversal sections (50 micrometer) along the rostrocaudal neural axis. We use the neuroanatomical terms for the adult zebrafish according to the zebrafish brain atlas by Wullimann and colleagues [25]. In these cases where we use the names of the anatomical structures to describe localization of expression pattern, *nestin *is often expressed at the ventricular surface of the named anatomical structures.

#### Telencephalon

**Figure 6:**
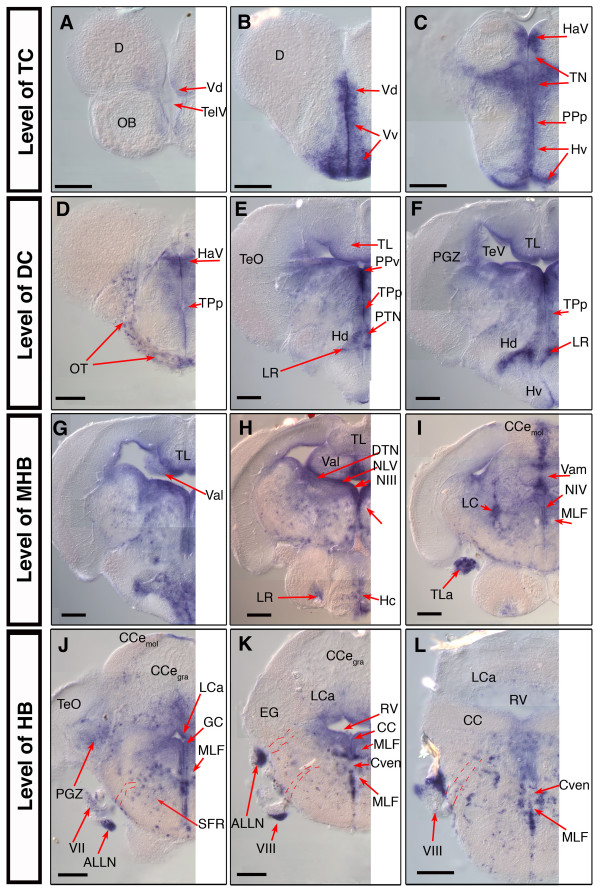
***nestin *expression in the juvenile 28 dpf old zebrafish brain**. Analysis of *nestin *expression in the brain of the 28 dpf zebrafish by whole mount *in situ *hybridization using *nestin *anti sense mRNA probes on whole dissected brain. (A-L): selected 50 μm cross sections from a serially sectioned brain, the rostrocaudal levels of the sections are indicated at left; dorsal up. Pictures show only the left half of the brain. Dashed lines in J, K, L indicate the tracts of the cranial nerves. *Abbreviations*: ALLN: anterior lateral line nerve; CCe: corpus cerebelli; Cven: commissura ventralis rhombencephali; D: dorsal telencephalic area; DiV: diencephalic ventricle; EG: eminentia granularis; GC: griseum centrale; HaV: ventral habenular nucleus; Hc, Hd, Hv: caudal, dorsal, ventral zone of the periventricular hypothalamus; LC: locus coeruleus; LCa: locus caudalis cerebelli; LR: lateral recess of the DiV; MLF: medial longitudinal fascicle; NIII: oculomotor nucleus; NIV: trochlear nucleus; NMLF: nucleus of MLF; NLV: nucleus lateralis valvulae; OT: optic tract; PGZ: periventricular gray zone of the optic tectum; PPa, PPp: parvocellular preoptic nucleus, anterior, posterior part; PPv: periventricular pretectal nucleus, ventral part; PTN: posterior tuberal nucleus; RV: rhombencephalic ventricle; TeO: optic tectum; TeV: tectal ventricle; TelV: telencephalic ventricle; TL: Torus longitudinalis; TLa: torus lateralis; TN: thalamic nuclei; Val, Vam: lateral, medial division of valvula cerebelli; Vd, Vv: dorsal, ventral nucleus of ventral telencephalic area; TPp: periventricular nucleus of the posterior tuberculum; III: oculomotor nerve; VII: facial nerve; VIII: octaval nerve; Scale bars: 100 μm.

The most anterior expression of *nestin *we could detect was a weak staining in the posterior part of the olfactory bulb (OB). Here *nestin *is expressed only in the ventral parts of the TC (V: ventral telencephalic area). Few *nestin *positive cells are detectable in the dorsal nucleus of the ventral telencephalic area (Vd) close to the telencephalic ventricle (TelV) (Fig. 6A). Throughout the TC *nestin *positive cells accumulate in the SVZ of Vd and Vv (ventral nucleus of V). Anterior to the anterior commissure (AC) *nestin *is strongly expressed in cells of the Vv and Vd, up to 5–10 cell diameters away from the ventricle surface (Fig. 6B). The expression is slightly reduced in the more posterior parts of the TC at the level of the AC. *nestin *is weakly expressed in the supracommissural and postcommissural nuclei (data not shown).

We also detected a weak expression of *nestin *in the ventral parts of the anterior parvocellular preoptic nucleus (PPa) (data not shown). At the level of the telencephalo-diencephalic boundary (preoptic region) *nestin *expression extends dorsally and is broadly expressed along the most anterior part of the diencephalic ventricle (DiV) in the area of ventral habenular nucleus (HaV), thalamic nuclei (TN), in the posterior parvocellular preoptic nucleus (PPp), and ventral periventricular hypothalamus (Hv) (Fig. 6C).

#### Diencephalon

*nestin *is expressed all along the subventricular zone of the DiV. In the region of the optic tract (OT) speckled *nestin *expression was visible, which might be glia precursor cells migrating along the optic tract (Fig. 6D).  Dorsally *nestin *is expressed in distinct ventricular zones: the ventral habenular nucleus (Hav), the periventricular pretectal nucleus (PP), the thalamic nuclei (TN) including the dorsal thalamic nucleus (DT) and the ventromedial thalamic nucleus (VM) (Fig. 6C,D). In the ventral DC *nestin *positive cells are detected in the region of the periventricular nucleus of the posterior tuberculum (TPp) and the posterior tuberal nucleus (PTN) (Fig. 6D,E). Strong *nestin *expression is detected in the cells adjacent to the lateral recess (LR) of the DiV in the area of the dorsal zone of the periventricular hypothalamus (Hd) (Fig. 6E,F) throughout its entire anterior-posterior extend (Fig. 6E–I). In the more posterior part of the ventral DC *nestin *expression is expressed at lower levels in the TPp and stronger in the ventral zone of the periventricular hypothalamus (Hv) (Fig. 6F,G). In cells adjacent to the DiV *nestin *expression is also detectable in the most ventroposterior part of the DC, in the region of the caudal hypothalamus (Hc) (Fig. 6H).

#### Mesencephalon

*nestin *is expressed in two zones of mesencepalon: in the optic tectum (TeO) and the torus longitudinalis (TL), which is ventrally attached to the tectum and reaches from its anterior to its posterior end [18] (Fig. 6E–G). *nestin *positive cells are located at the ventricle-contacting surface of the torus longitudinalis along its entire anterior-posterior extension in the mesencephalon (Fig. 6E–G). Further, in the TeO cells of the ventricle contacting part of the periventricular gray zone (PGZ), express *nestin *mRNA in a relatively homogeneous manner around the ventricle. The expression domain spans the entire rostro-caudal axis of the PGZ. The more superficial layers of the TeO and the parts of the PGZ not contacting the ventricle do not express *nestin*. *nestin *is also strongly expressed in the dorsal tegmental nucleus (DTN) and the nucleus lateralis valvulae (NLV), two ventricle contacting structures of more ventral parts of the mesencephalon (Fig. 6H). In the most rostral part of the superior reticular formation, which also belongs to the mesencephalon, *nestin *expressing cells can be detected in a scattered manner (Fig. 6J).

#### Hindbrain and midbrain hindbrain boundary (MHB)

In teleosts the cerebellum can be subdivided in three parts [16,25]: The corpus cerebelli (CCe), the vestibulolateralis lobe (consists of the medial caudal lobe (LCa) and the eminentiae granulares (EG) and the valvula cerebelli pars medialis and pars lateralis (Vam/Val). The midbrain hindbrain boundary (MHB) is known as a zone of continuous neurogenesis in the developing as well as in the mature brain. The MHB in the post-embryonic brain also contains *nestin *expressing cells. In the anterior part of the cerebellum *nestin *is strongly expressed along the midline, mainly in the molecular layer, the granular layer seems to be devoid of *nestin *expression cells. (Fig. 6H,I). *nestin *expressing cells can be found in both, medial and lateral, parts of the valvula. In the Val we could detect strong *nestin *expression in ventricle contacting regions. In the medial part of the valvula cerebelli (Vam) *nestin *is strongly expressed in the molecular as well as in the granular layer close to the tectal ventricle (Fig. 6G–I). In the eminentiae granulares *nestin *expressing cells are detectable. In the HB like in other parts of the brain, *nestin *expressing cells are located in the SVZ of the rhombencephalic ventricle. Here strong expression is detected in the ventricle contacting part of the caudal lobe of the cerebellum (LCa) (Fig. 6J–L). Further, in the HB more widely distributed *nestin *positive cell groups, e.g. in the granular and molecular layer of the caudal lobe, are detectable compared to the more anterior parts of the brain (Fig. 6J–L).

#### Cranial Ganglia Nerves

In the post-embryonic brain as well as in the embryonic brain, *nestin *is expressed in the nuclei of cranial nerves. High expression of *nestin *is detected in the nucleus of the third cranial nerve (oculomotor nerve; NIII) (Fig. 6H). The axon-containing tract of the medial longitudinal fascicle (MLF), which carries axons descending to the spinal cord, is devoid of *nestin *expression throughout its rostrocaudal extend (Fig. 6H–L), as well as in the nucleus of the trochlear nerve (nervus trochlearis, IV) (Fig. 6I). Surprisingly, in the area of cranial nerves, which have already left the brain stem, we detected strong *nestin *expression. Analysis at higher resolution revealed that *nestin *is not expressed in the fascicles themselves but in clusters of cells, which appear to accompany the nerves. This was observed for the fascial nerve (n. fascialis, VII) as well as for the octaval nerve (n. octavus, VIII) and the associated anterior lateral line nerve (ALLN), and led us to speculate that these cells might be glia or migrating glia precursors, similar to findings in mammalian systems [26] (Fig. 6G,J,K,L).

## Discussion and Conclusion

In mammals the intermediate neurofilament Nestin is a well established marker for neuronal stem cells and proliferating precursor cell populations, as well as for precursor cell populations in some other tissues (reviewed in [5]). Previously, *nestin *expression has only been characterized in mammalian systems [2,10,11], and chick [12]. However, while a gene prediction for a zebrafish *nestin *has been placed by ENSEMBL on linkage group 16 at 27.11 Mb, until now it has not been demonstrated that this intermediate filament represents a true *nestin *ortholog in teleosts. Here we provide two lines of evidence that this zebrafish intermediate filament is the true *nestin *ortholog: (1) Zebrafish Nestin protein sequence clusters with higher vertebrate Nestin proteins, and is clearly separated from other vertebrate intermediate neurofilaments, based on phylogenetic tree analysis. (2) *nestin *is expressed at embryonic, larval, and juvenile stages in cell populations which in the CNS largely represent stem and precursor cells. Subsets of *nestin *positive cells co-express the proliferating cell nuclear antigen PCNA – zebrafish *nestin *expression thus correlates with the expression pattern described in mouse [4].

We found that outside of the nervous system *nestin *is expressed in mesodermal muscle precursor cells and in craniofacial mesenchyme. This correlates with *nestin *expression in mammals at comparable developmental stages, which has been reported for head mesenchyme and muscle precursors [6]. In contrast to the reported *nestin *expression in mouse, we could not detect *nestin *expression neither in developing somites nor in the myotome of more mature somites at any stage. Our analysis was focused on *nestin *expression in the nervous system, and therefore we did not test whether there may be correlates to *nestin *expression reported in mammalian epidermis, heart, pancreas, kidney, and lung (reviewed in [5]).

During late somitogenesis stages zebrafish *nestin *is widely expressed throughout the developing nervous system. A similar widespread expression has been reported for comparable stages of mouse development where *nestin *expression and protein distribution were investigated by *in situ *hybridization and immunohistochemistry [1,3]. Further, the comparison of zebrafish *nestin *expression with GFP expression in nestin promoter-GFP transgenic mice, which has been described in great detail [4,27,28], strengthens the notion of evolutionary conserved roles of Nestin in stem and precursor cell development. With progressive development of the nervous system in zebrafish, like in mouse, *nestin *expression becomes gradually restricted to regions of the CNS which have previously been identified as zones of proliferating stem and precursor cells: the ventricle walls in the CNS, the ciliary marginal zone in the retina and some scattered cells in the medulla oblongata [16-18].

In the peripheral nervous system *nestin *is strongly expressed in cells associated with the octaval ganglion, the lateral line ganglia, the facial and trigeminal ganglia, and the ganglia of the nervous vagus. It has been reported for mice that *nestin *in the PNS is expressed in astrocytes associated with neurons of the cranial ganglia [26], therefore it is likely that the *nestin *expressing cells associated with zebrafish ganglia are also glia.

The zebrafish brain grows throughout post-embryonic stages as well as in mature adult fish. To analyze *nestin *expression in the maturing post-embryonic and post-larval brain, we studied the brain of four weeks old zebrafish. In the maturing nervous system *nestin *expression was detected in all areas, which were recently described as stem cell niches and zones of proliferating precursor cells [18]. In the forebrain, these are: the ventricular zones of the tel- and diencephalon, the ventral habenular nucleus (Hav), the periventricular pretectal nucleus (PP), the thalamic nuclei (TN) including the dorsal thalamic nucleus (DT), and the ventromedial thalamic nucleus (VM). In the mid- and hindbrain, these are: the mesencephalic areas of the torus longitudinalis and optic tectum adjacent to the ventricle, the MHB, proliferative zones in the cerebellum, and ventricular zones of the hindbrain and the spinal cord. In summary, *nestin *expression in the maturing, post-embryonic CNS correlates with the previously described proliferation zones [16-18]. Thus, the expression of the intermediated neurofilament *nestin *in zebrafish, like in mammals, may serve as a marker for stem cells and proliferating precursors in the developing embryonic nervous system as well as in the adult brain.

## Methods

### Fish

Zebrafish (AB strain) breeding and maintenance was under standard conditions at 28.5°C [29]. Zebrafish embryos (AB strain) were staged and fixed at the desired developmental stages according to Kimmel et al. [30]. To inhibit pigmentation embryos were incubated in 0,2 mM phenylthiourea (Sigma).

### *In situ *hybridization and histological sections

Whole-mount *in situ *hybridization (WISH) was performed as described [31]. A Digoxigenin-labeled antisense mRNA probe for *nestin *was used. For generation of the *nestin *probe we amplified a fragment from genomic DNA with primers 5' nes_1F: GTACCAGATGCTAGAGCTGAACCACCGCCTTG; 3' nes_1R: GCATCTGCCTCTTGATCCTCGTGCTCTCCAG. These primers amplify a 704 bp fragment of the second exon of the *nestin *gene (ENSEMBL gene prediction ENSDAR00000040236), which we cloned it into the pBSII-KS vector. For sectioning, embryos were embedded in a mix of 0,5% gelatin, 30% bovine serum albumin, and 20% saccharose dissolved in PBS. Polymerization was initiated by adding 70 μl of 25% glutaraldehyde per 1 ml, the embryos were oriented, and polymerization completed by addition of another 70 μl of 25% glutaraldehyde. Blocks were mounted with glycergel, and 50 μm sections were prepared with a Leica Vibratome.

Double fluorescent whole-mount *in situ *hybridization (FISH) for *nestin *and *pcna *was performed modified from [32,33], which were adapted to zebrafish (Alida Filippi and Wolfgang Driever, unpublished, and (30)). Confocal images were recorded with a Zeiss LSM 5 DUO laser-scanning confocal microscope.

For analysis of *nestin *expression in the brain of 28 dpf fish, animals were anesthetized with tricaine before they were killed in ice water. The brain was dissected out and fixed in 4% paraformaldehyde/PBS/0,1 % Triton X-100 over night at 4°C. In situ hybridization was performed as reported for whole mount 24 hpf zebrafish embryos [31]. After the staining, brains were embedded in 3% agarose/PBS and serial sections (50 μm) generated using a vibratome.

## Abbreviations

ALLG: anterior lateral line ganglion

ALLN: anterior lateral line nerve

CC: crista cerebellaris

CCemol/gra: corpus cerebelli, molecular, granular layer

CeP: cerebellar plate

Ch: chorda dorsalis

cm: craniofacial mesenchyme

CMZ: ciliary marginal zone

Cven: commissura ventralis rhombencephali

D: dorsal telencephalic area

DC: diencephalon

DiV: diencephalic ventricle

DT: dorsal thalamus

DTN: dorsal tegmental nucleus

EG: eminentia granularis

eg: enteric ganglia

eth: ethmoid plate

FB: fin bud

FG: facial ganglion

FP: floor plate

g: gut

GC: griseum centrale

GCL: ganglion cell layer

Ha: habenula

HaV: ventral habenular nucleus

Hc, Hd, Hv: caudal, dorsal, ventral zone of the periventricular hypothalamus

Hi: intermediate hypothalamus

hm: head mesenchyme

LC: locus coeruleus

LCa: locus caudalis cerebelli

LR: lateral recess of the DiV

MHB: midbrain hindbrain boundary

MLF: medial longitudinal fascicle

MO: medulla oblongata

NIII: oculomotor nucleus

NIV: trochlear nucleus

NLV: nucleus lateralis valvulae

NMLF: nucleus of MLF

no: notochord

OB: olfactory bulb

OC: otic capsule

OE: olfactory epithelium

OG: octaval ganglion

OT: optic tract

pc: parachordal cartilage

PGZ: periventricular gray zone of the optic tectum

Po: preoptic region

PPa, PPp: parvocellular preoptic nucleus, anterior, posterior part

PPv: periventricular pretectal nucleus, ventral part

pq: palatoquadrate

Pr: pretectum

PTN: posterior tuberal nucleus

PTv: ventral part of the posterior tuberculum

Ret retina

RV: rhombencephalic ventricle

S: subpallium

SC: spinal cord

SFR: superior reticular formation

som: somites

T: midbrain tegmentum

TC: telencephalon

TelV: telencephalic ventricle

TeO: optic tectum

TeV: tectal ventricle

TG: trigeminal ganglion

TL: torus longitudinalis

TLa: torus lateralis

TN: thalamic nuclei

TPp: periventricular nucleus of the posterior tuberculum;

TS: torus semicircularis

Val, Vam: lateral, medial division of valvula cerebelli

Vd, Vv: dorsal, ventral nucleus of ventral telencephalic area

VG: vagal ganglion

VMP: ventral motoneuron

III: oculomotor nerve

VII: facial nerve

VIII: octaval nerve

## Authors' contributions

JM performed all experiments, assembled the figures, did the sequence alignments, and wrote a first draft of the manuscript. WD conceived the study and participated in the experimental design, initially identified zebrafish *nestin *orthologous sequences, and helped to draft the manuscript. All authors read and approved the final manuscript.
